# Extra Supplement—The Nursing Mirror

**Published:** 1889-03-16

**Authors:** 


*The Hospital,*
MARCH 16, 1889.

### The Nursing Mirror.

[*Extra Supplement.*

All Contributions for this Supplement should be addressed "The Nursing Mirror," care of The Editor, 38, Old Jewry, E.C.

### En Passant.

NATIONAL PENSION FUND FOR NURSES.—At a meeting of the Council held on the 7th inst., Mr. Walter H. Burns in the chair, the Honorary Manager reported that the number of applications for pensions and sick pay up to date was 725 (exclusive of the Mildmay nurses and the nurses of the Seamen's Hospital, numbering about 133), of whom 567 had paid their contributions, amounting to £10,774, of which £714 had been received since the last monthly meeting. Thirty-one applications had been received during the corresponding period, and twenty-six were accepted at the present meeting. It was reported that the funds invested amounted to upwards of £34,500.

SCOTCH NURSING.—Far away in the North of Scotland the influence of Florence Nightingale and her successors is slowly being felt. Lady Mackenzie is now starting a District Nursing Society at Dingwall and the surrounding parishes. The main feature of this association is that it is a "benefit society," which entitles all who subscribe to it regularly to the services of a sick nurse when required at a reduced fee. The nurses employed undergo a modified training, which has been found sufficient. They are engaged by the association, and their duties are assigned to them by a committee or by the secretary, to whom applications for nurses have to be addressed. The members of the association (who belong to all classes within the district), pay subscriptions at different rates, according to their circumstances in life, and the fees for a nurse's services are on a scale which varies in the same manner.

NURSE AND WATCHER.—The lady who wrote the first article in the *Jewish Chronicle* on the subject of Jewish nurses has written a second one, in which she deals with suggestions since afforded by others, and makes a sensible suggestion of her own. The last is that a trained nurse should be connected with every synagogue. It is impossible for Christians to comprehend why a nurse should also be a watcher, but two pictures drawn in this article largely explain matters. This is the first: "The watcher has been hastily summoned, and stands beside the bed, and as the poor sufferer's eyes rest on her they cast an appealing look to the group of sorrowing relatives, as if asking that the gloomy herald of death may be removed. And so the watcher is placed behind the curtains, but ever and anon some movement denotes her presence, and seems to make the dying patient shudder. At length the moment of death comes, and the last prayers are hurriedly muttered by the watcher who has been summoned from her daily avocation to come to pray with the passing spirit. All is over now, and the weeping relatives are called on to leave the form of their dear one to the rough hands which in the sufferer's lifetime would not have been allowed to approach." And this is the second: "See the calm, gentle woman that is performing tender offices for the sufferer and speaking peace and consolation; hear the holy words of solace she addresses to the grieving relatives. And now the solemn prayers for the dying are offered up slowly and reverentially by lips accustomed to move prayerfully. The end comes at last, and gently and kindly the nurse leads the mourners away from the sad scene. They can trustfully leave their dead with the one who has shown so much tenderness while life was still present, and who has helped so greatly to smooth the path into the shadows." There is one suggestion to which the writer of these articles does not condescend to reply, and that is the suggestion of forming a committee, which we denounced last week as absurd and intrusive.

PENSIONS FOR READING NURSES.—The committee of the Royal Berkshire Hospital are determined that their private nurses shall receive the full value of their earnings, and that their labour shall not go to support the hospital expenses. They have therefore formed a pension scheme on these lines:—The annual balance of profit on the private nurses' department to be invested, as a separate reserve fund, for the security of the scheme. Twenty per cent. of each nurse's quarterly salary is deducted, and placed in her behalf in the Reading Savings Bank, and there permitted to accumulate. On completing four years of service an annual bonus of 10 per cent. on her salary, and on completing eight years of service a further annual bonus of 10 per cent. is added to her trust account; the whole sum accumulating until she quits the service of the hospital, or for certain purposes, and with the permission of the trustees, requires to withdraw her trust fund. If, however, she remains in the service of the hospital till the age of 55, the trustees, with her assent, will purchase for her, with the whole or some portion of her trust fund, a Government annuity. Such, omitting minor details, is the pension scheme, and the board feel that the nursing staff has thoroughly deserved this recognition at their hands. Again and again the most gratifying testimonies to the devotion and efficiency of both private and district nurses have been received. All will agree that the nurses must thank the committee for dealing thus honestly and kindly with them. We scarcely see, though, how the nurses can afford to save 20 per cent. of their income; if the fund were affiliated to the National Pension Fund, a smaller percentage would secure all that is necessary. When we have a national fund so generously endowed, it ought to secure the support of all hospitals and nurses, and smaller funds should be merged into it for the good of all concerned.

LADIES AS NURSES.—The correspondence on this subject in the *Standard* has been growing larger and more ridiculous every day. A whole column of letters appeared last Saturday, some of them sensible and interesting enough, but others lacking alike in knowledge or composition. The general opinion expressed is that hospital authorities should make the nursing work easier; and, as a matter of fact, they have been doing this lately in all good hospitals. Since ladies have taken to nursing, the old rules framed for the domestic-servant class have had to be revised, but it would be absurd to introduce such laxness as many seem to desire. Ladies who become nurses must remember that by so doing they become working women, and that only by order and method can good work be thoroughly done. The leisure, unpunctuality, and *dilettanti* habits which make home life pleasant, are impossible in a hospital; and a lady who is not satisfied to be a working woman is not fit to be a nurse; her proper sphere is the drawing-room, not the ward. We look forward for more holidays and higher wages for nurses in the future; because we look back and see how greatly their duties have been lightened and their lives made easier during the last few years. We would like to quote one letter from the *Standard*: "'A Six Months' Probationer' says she has never at home enjoyed such perfect health as she did during the six months she spent at an important provincial hospital. A friend of hers—a very delicate girl—found the work very hard and very trying; but she was well and happy most of the time. After her year's training, she went home, and now her letters tell of fatigue and low spirits, following upon late hours and the veritable hard work which a girl, fond of society, will go through at any cost. One party she 'had to leave early, quite exhausted'; and those who do not go in so much for the gaieties of life fill up their time with other voluntary work which is quite as irregular. This correspondent thinks that the regular hard work of hospital life is less trying than the irregular hard work of many girls who live at home."

### Religious or Lay Nursing?

### THE QUESTION IN PARIS.

At last the decree which banished the Sisters of Mercy from the Parisian hospitals has been repealed, and these noble women are free to resume their old place in the wards where they reigned supreme for so many years. On the last day of February the Sœurs de Charité were to have been banished from the Quinze-Vingts Hospital, almost the last place where they still did the nursing; but the new Government came into power just at the right time to prevent this eviction, and to make it law for the Sisters to nurse wherever their services were asked and wanted. The question of religious and lay nurses is one which has numerous *pros* and *cons*. It was just as unjust for there to be no lay nurses in the last century as it is for there to be no religious nurses in the present time; and doubtless the Sisters will have learnt much through their late tribulation. The Sisters had been so long the sole nurses in Paris that they had come to be didactic in their practice and conservative in their theories. They did not relish the new style of training the doctors tried to introduce; the lectures, bandaging classes, and certificates. They put their faith too much in gentle but untrained service, and in prayer rather than in knowledge. Their clumsy woollen dress was unfitted for sick-nursing, especially infectious cases; and their narrowness of thought gave monotony to the hospital wards. But their devotion was unswerving, their love untiring; body and soul they gave themselves to their labours, and no patient could ever complain of inattention or lack of sympathy while they reigned in the wards.

The first introduction of lay nurses was hailed with joy. The doctors felt that these women would be more teachable; the patients, that they would be more cheerful. But the system was new, and the nurses were a bad class of women and not under proper control. There was no one to train them, the Sisters being banished, and silently and sorrowfully lay nursing was acknowledged a failure. Cases of inattention were common, bribes and presents were taken by the nurses, several serious accidents happened to patients through carelessness or ignorance, and the last state of nursing was worse than the first. During the transition this was only to be expected, and lately the lay nursing in several of the Parisian hospitals has been highly praised; still it is pleasant to think that the Sisters are not banished for ever from their ancient haunts. One scarcely sees how it could be worked, but if the two forms of nursing could be amalgamated, keeping the good of both and leaving the evil, the Parisian hospitals would have well solved the question at last. Six religious communities were formerly responsible for the nursing in Paris. The Augustines reigned at the hospitals of La Charité, Saint Louis, La Riboisière, and L'Hôtel Dieu. The Sisters of Sainte Marthe nursed at Saint Antoine, La Pitié, and Beaugon. The Sisters of Saint Vincent-de-Paul at Necker and Sainte Eugénie, the Sisters of Compassion at Lourcine and the Sisters of Sainte Marie at Cochin, and the sisters of Saint Thomas at the Enfants Malades. They were always gentle, and liked by the patients, though they must often have been severely tried by the cases at the Lourcine and elsewhere. When the *Assistance Publique* first introduced lay nurses to the number of 491, they were recruited from domestics out of place, dismissed shop-girls, and a like class. They were paid from 15 to 20 francs a week and given lodging, food, and uniform; but even at this wage they were not angels. They drank the patients' wine; they demanded tips; and they are even accused of having drunk the spirit in which the doctors preserved their specimens! Yet these nurses were all true to their posts in 1870, when an epidemic of small-pox passed over Paris, and, doubtless, under a proper system of training might be greatly improved. Since Parisian ladies are not likely to take to nursing as English ladies have, it is just as well that the gentle and cultivated Sisters should still exercise their influence in the hospital wards. It may interest our readers to know that the Hôtel Dieu contains over 800 beds; La Pitié over 700 beds; La Charité over 400 beds; Enfants Malades over 600 beds; La Maternité over 300 beds. In 1873 the 15 hospitals of Paris treated 83,000 in-patients, and 7,500 sick persons were in the hospitals on December 31st of that year.

### Nursing Sisterhoods.

### THE COMMUNITY OF ST. JOHN THE DIVINE.

When we read accounts of the old contemplative Religious Orders of the Middle Ages, and then turn to the consideration of our active communities of these modern days, we cannot but believe that progress has here done as good and as marked work as in all other lines of life. The nun of olden days spent her time in an unceasing round of prayer and self-introspection and self-discipline; in fact, self was the point on which all her actions turned. Her meditations and mortifications were alike devoted to the saving of her own soul, and for those without the convent gates she had neither thought nor charity. Different, indeed, is the life in a modern Sisterhood, where worship is expressed by work, and where deeds are a ceaseless prayer to God.

The report for 1888, just issued by the St. John the Divine Community, is an account of active charity towards all sorts and conditions of men. The first aim of the community is to ensure the supply of nurses of skilled training and high moral character to rich and poor alike. As a proof of what a band of devoted women can do, it is a truly inspiriting report.

There are eight different centres from which the work of the Community is carried on: First there are the head-quarters, at 68 and 70, Drayton Gardens, where the Sister Superior lives, and from whence trained nurses are sent out to private families. Here also ladies should apply who desire a three months' training to fit them for meeting emergencies in their own families. Two or three private rooms are also reserved at 70, Drayton Gardens for paying patients under the care of their own doctors, the fee to the home being six guineas a-week.

At Gunter Grove, Chelsea, there is a lying-in hospital, first opened in a small way in 1877, and now consisting of three detached houses and a nice garden. During 1888 there have been admitted 140 women to this hospital, and 160 have been attended at their own homes. The household expenses during the year have been £666 14s., not including rent, taxes, insurance, and repairs; with these latter items included, the total expenditure amounts to £927 14s. 9d. It will be evident what a heavy tax the almost entire support of the home—the whole amount of donations and subscriptions being £81—is upon the community, and they do earnestly beg for the support and donations of their friends, and especially that of mothers. The great object of this work was that of providing a comfortable lying-in home for the benefit solely of respectable married women amongst the very poor. Chelsea being a densely-populated district, afforded a wide field for operation in this respect, and the special features of the home are much appreciated by those for whose benefit it was founded. The chief of these are that only married women are admitted; that there are no wards, but each woman has a separate room; and that only a nominal charge of 1s. is made for booking.

At Montague Place, Poplar, the Sisters have a district home, where nurses reside who attend to the poor in their own homes. During last year, 914 separate cases have been nursed for longer or shorter periods, varying from a few days to two or three months or more, and in many instances by night as well as by day. An experienced midwife attends upon the women in their confinements, and has been called to 202 such cases in 1888, making a total for the year of 1,116 cases, and since its opening of 7,394. A class for factory girls is also held here, and other means of doing good are carried on.

Next the district home is St. John's Hospital for Women and Children, which we visited lately and found a very cosy and cheerful little hospital. It was opened in 1884 by the Earl Nelson, and is quite free to the poor of the neighbourhood. Last year 156 cases were treated, and the total expenditure was just over a thousand pounds.

The largest hospital in charge of the Sisterhood is that at Lewisham, which is for both men and women. Here again there are paying wards, the charge being from two to three guineas a-week. Only last week a sad death from burning was recorded in the daily papers as having taken place at this hospital. A woman blew down a paraffin lamp, which exploded and set her clothing on fire. To have a hospital to which to take such painful cases must be a great boon to the neighbourhood. The hospital has a large garden, which looks very pretty in summer, and is greatly enjoyed by semi-convalescents.

The last hospital nursed by the Sisters is perfectly unique of its kind—St. Gabriel's Hospital for Infants under two years of age. Fancy 114 of these tiny sufferers being cared for last year! The very notion of such wee mites needing nursing should call forth all our pity. The hospital is generously supported by a lady, and is no expense to the community.

But hospitals are poor affairs without that useful adjunct, a convalescent home; and so a pretty cottage at Littlehampton opens its doors to those discharged from the above hospitals. To all, this seaside home has been an unspeakable pleasure, and the vicinity of Arundel Castle has added to the enjoyment. Several of the nurses get a holiday and a rest here also, when the hard work of private cases, or the constant wear of hospital duties, has left its mark on their health. This home is also supported by the generosity of a single lady, towards whom all the patients express their deep gratitude.

That is the total—four hospitals, two district homes, one convalescent home, and head-quarters for supplying private nurses. This is a record of work not to be despised, and we honour the Sisters of St. John the Divine for what they have done, and hope their sphere may be yet further widened and enlarged.

### WORK AT DURHAM.

THE Durham County Hospital Samaritan Fund supports two nurses, who do good work in the town. The report of their work for last year is as follows: 264 patients have been attended; of these 140 were district cases, and 124 hospital; 7,034 visits have been paid; 244 orders for meat and 498 orders for milk have been given; and beef—to the value of £3 18s.—has been made into beef-tea, and given to 316 patients; wine and spirits—to the value of £6 6s. 6d.—have been given, always by the doctor's orders, and as part of the medical treatment. This society is appealing for further subscriptions, and surely it deserves them, for this is what the house surgeon of the hospital says about it: "I have closely observed the work of the Samaritan Society among the out-patients of this hospital, and am more convinced than ever of its value; the kindly and skilful attentions of the nurses; the timely aid, in the shape of milk, beef-tea, and other necessities of an invalid diet; the residence at the seaside afforded to the debilitated convalescent, are all evidences of the excellence of the work. To give only one instance: In one family, living in two small rooms, the father and five of his children were laid low by typhoid fever, and all the cases were attended to a successful issue by one of the nurses of the society. No reference to the work would be complete without referring to the fact that patients are assisted in getting artificial limbs after amputation, instead of being discharged from the hospital helpless cripples."

### Nursing the Insane.

THERE is one branch of nursing in which England is far behind America and the Colonies, and that is in the training of attendants for the insane. There can be no doubt that insanity is one of the most awful maladies to which humanity is subject, and one of the most mysterious and most difficult to treat. The insane person must often suffer tortures from the uneducated attendant who too often has charge of such cases; with nerves disordered and mind unhinged to be put completely in the power of a tactless, untrained man or woman must be only too likely to increase the patient's malady. Every large asylum ought to have its training school, its lectures, and its certificates, just as every large hospital has. The nurses at Philadelphia and Buffalo are taught to give intelligent service to the insane, but not so the nurses of London or Edinburgh.

This question lately came forward in Australia at the Medical Congress, when Dr. Williamson read a paper on "Training of Asylum Attendants and Nurses." He gave an interesting description of what was being done in this way in Australia, and gave it as his opinion that the nurses and attendants who were trained at Gladesville, New South Wales, had displayed a knowledge and ability which was as creditable to themselves as to the medical officers who taught them. The whole subject was, he thought, only in its infancy. There could be little doubt of its growth and extension. He believed that the old order of things would soon give way, and that a few years hence would witness in each asylum for the treatment of the insane, in Australia, the establishment of a regular system of training, both for nurses and attendants. He knew of no one subject in the special branch of medicine treated in the section which was fraught with more important issues to the welfare of patients, or which bore more closely on the actual success of medical officers in ward work. The far-reaching influences arising therefrom could not at present be estimated, and new possibilities of all kinds would open out under the influence of such a staff as might be obtained when nursing was taught in asylums as fully and completely as in other hospitals.

Another paper, read by Dr. Eric Sinclair, said there was a need for improved nursing arrangements in asylums, and a nursing staff should have systematic training. There should be a ward large enough to contain 40 or 50 patients, and separate from the main hospital. All new admissions should be sent there, as well as all cases of sickness occurring among the ordinary patients. Thus attendants or nurses would have an opportunity of seeing all kinds of illness, bodily as well as mental. The main use of the ward would be the training of the staff. A further extension of hospital methods that might with advantage be made was the training of nurses and attendants for private work. In America it had been found possible to institute a training school for attendants on the insane apart from the asylum staff, and we should be able to do the same in such a ward. Another hospital method which might be adopted was the separation of the nursing from the cleaning housemaid and labouring work, this being found necessary to induce well-educated and gently-nurtured women to become nurses.

All who have read Dr. C. K. Mills' interesting book on the "Nursing and Care of the Nervous and Insane," and have seen how strongly he recommends more intelligent and more highly-taught nurses for asylums, will hope that this subject may shortly come to the fore in England. There are some well-trained mental nurses here—at St. George's Retreat, Sussex, for instance, the sisters are excellent attendants—but it should be recognised that it as necessary for an asylum nurse to be trained, as for a hospital nurse.

### Everybody's Opinion.

### FAIR OR NOT?

A. H. R. writes:—Will you, or any of the readers of The Hospital, inform me if it is usual when a nurse is sent for by her friends in a case of emergency, for the Hospital authorities to compel her to find a substitute or the equivalent of a substitute in money? Few nurses can afford this, and it seems that a nurse who gives up the best part of her life to her work, should also have to give up her friends and relatives when her presence is most needed by them. A case has lately occurred in a large hospital in the West of England where a nurse was sent for by her friends, and to whom leave of absence was granted, only on condition that she should pay 25s. that a nurse might be obtained to fill up the gap her absence would cause. The nurse went, and the following day sent the money which was duly acknowledged by the secretary.

As a matter of fact no extra nurse was obtained, but a probationer was talen from a staff nurse already over-worked with ward and other duties, and put in the place of the absent nurse. Hence arises another question, what should be done with the 25s. acknowledged by the secretary?

Apologizing for taking up so much space in your valuable paper,

### Lectures.

At the Sanitary Institute, on March 26th and 29th, April 2nd, 5th, 9th, and 12th, lectures will be delivered at three p.m., on Domestic Hygiene. Fee for the course, including examination, 10s. The first lecture will be on "Child Culture," by Dr. Schofield; the second and third on "Dairy Produce," by Wynter Blyth; the fourth on "The Chemistry of Cleaning," by Professor V. B. Lewis; the fifth on "Bread," by W. Jago; and the last on "Petroleum Products," by Boverton Redwood. The lectures will be illustrated by diagrams, specimens, experiments, and microscopical demonstrations. Tea will be served after each lecture, when the lecturer will gladly answer all questions. Her Royal Highness the Duchess of Albany has graciously expressed her intention of personally patronising the lectures. An examination will be held after the lectures, and certificates granted to successful candidates.

At St. Stephen's, South Kensington, during Lent, Rossini's *Stabat Mater* will be sung on Friday evenings, at eight o'clock, with a full orchestra. Dr. Stainer's oratorio, *The Crucifixion*, will be performed on Sundays, at four p.m.

### Presentations.

Miss Irene Hardy, the honorary lady superintendent of the West London Hospital, has been presented by the committee of that institution with a gold watch and chain, of the value of £50, as a token of their appreciation of her kindness, attention, and zeal.

### A NURSES' SAVINGS FUND.

An Army Sister writes:—

Having seen the letter signed "Handclasp" in the Nursing Supplement to The Hospital for January 26th, I hope I may be pardoned the liberty of saying with what great interest and pleasure we nurses here have read it. We are most anxious to join the Pension Fund, and do thoroughly appreciate the nobility and generosity of those who have started such a fund. What some of us feel about it is this: Were it a bank in which nurses might safely deposit their earnings, receiving at the end of each year the interest on the same (say 5 per cent.), which could again be deposited by themselves if wished, the whole being allowed to accumulate towards a pension, then each nurse might have her own savings bank book made up yearly, she would know exactly what she had deposited, and should she choose to withdraw it at the age of fifty, the whole amount might then be supplemented by the bonus promised now by the Pension Fund. It seems to us much less like charity and more like being independent, to pay what one can each year, getting interest for the same, than to rely on the bounty of the Council to make good the annual deposit one may not always be able to pay.

[We shall be very glad to have the opinion of as many engaged in nursing as possible upon this proposal of an Army Sister. A post-card containing the above heading, with the words "I approve," or "I disapprove," and the name and address of the nurse who sends it, will be of great service if forwarded to the Editor, The Hospital, The Lodge, Porchester Square, W., within the next ten days. It is hoped that as many nurses as possible will thus record their votes on this important suggestion. The interest could never, probably, exceed 2½ per cent. on such deposits.—Ed. *T. H.*]

### Appointments.

Glasgow Royal Infirmary.—Miss Elsie Thorn, night superintendent of medical nurses in the Glasgow Royal Infirmary, has been appointed matron of Ayr County Hospital, vice Miss W. Ewin, resigned. Miss Thorn was trained at the Queen's Hospital, Birmingham, under Miss Stephens, and has held her present appointment for nearly two years. We congratulate Miss Thorn upon her rapid promotion, and wish her good speed.

Nurse Morton (Guy's Hospital) has been appointed matron of the Sidcup Cottage Hospital.

### Vacancies.

The following vacancies are announced:—

**Matrons.**

Princess Alice Memorial Hospital, Eastbourne; £50.

**Superintendent of Nurses.**

City of Birmingham Scarlet Fever Hospital.

**Nurses.**

Brecon Infirmary; £18.

Eastbourne Nursing Institution.

West Bromwich Hospital; £22

73, Wharton Road, Kensington.

St. John's Institution, Southport.

Hertford Infirmary; £20.

Kingston Home, Hull.

County Hospital, Guildford; £18.

Plymouth Private Nursing Home.

Hospital for Consumption, Broughton.

Denbighshire Infirmary; £25.

Ryde Nursing Institution; two.

Barrow in Furness; £20.

Royal Westminster Ophthalmic Hospital.

Cornwall Trained Nurses' Home; £25.

General Hospital, Tunbridge Wells; £25.

Bedford Nurses' Institute; £25.

Deaconess House, Chester; £25.

Stroud Hospital (night); £20.

Southport Trained Nurses Home.

Bechenham College Hospital.

Worcester City and County Institution.

Darlington Fever Hospital; £26.

Levich Paris Institution.

Barnhill Hospital, Glasgow, £24.

Bolton Infirmary.

Oakfield Home, Forest Hill.

Birmingham Workhouse Infirmary.

Public Hospital, Sheffield; £22.

Tewkesbury Hospital; £20.

Nottingham and Notts Nursing Institution; £25.

Wimbledon Nursing Institution; two.

Walsall Cottage Hospital.

Institution of Trained Nurses, Leicester; four.

**District Nurses.**

Shalford, Guildford.

Cheltenham District Nursing Association.

Bolton.

Ealing Dean.

Camborne, Cornhill.

Walton-on-Thames; £26.

**Probationers.**

Halifax Borough Hospital.
Boscombe College Hospital.
Barrow in Furness; £16.
Royal Surrey County Hospital, Guildford; £12.
Cromer College Hospital.
Bolton Infirmary.
Tewkesbury Hospital.
Sherborne Hospital, Dorset.

**Wardmaids.**

Cromer Cottage Hospital; £10.
Hove Fever Hospital; £16.

### Notes and Queries.

**Queries.**

(127) *Macintosh.—Kittifonia* would be glad if any nurse who has worked in the tropics would tell her whether there is any sort of macintosh that stands the climate, and if so where it is to be procured. If not, what is used as a substitute (*a*), for operations and the protection of bedding, (*b*) for covering poultices, dressings, etc.? her own experience (not as a nurse) being that all kinds of macintosh, American cloth, etc., speedily become sticky, hard, and useless.

(132) *Hospital in Paris.*—Can anyone tell me of a hospital or institute in Paris where a knowledge of French is not required?—*Nurse W.*—[The Hertford Hospital.]

(133) *Home for N rses, Melbourne.*—Can anyone give *Nurse Agnes* any information about the Home for Nurses in Melbourne—if it is a comfortable one, if there is a good chance of a trained and competent nurse getting work there, and what is the name and address of the superintendent?

**Answers.**

(119) *Soap.*—I have found Sunlight soap cheap, cleansing, pleasant, and not in the least irritating to the skin. Its only fault is that it washes away quickly.—*Experientia.*

(120) *Medical Dictionary.*—By applying to H. Kimpton, medical bookseller, 82, High Holborn, W.C., a *Private Nurse* will obtain a recent and well-preserved copy of *Hoblyn's Dictionary* (second hand) at about 4s. 6d.—*A. B.*

(123) *Naval Nurses.*—You must train two years in a general hospital, and then apply to the Under Secretary of State for War.

(124) *Home Wanted.*—Many homes are mentioned in the "Englishwoman's Year Book," Hatchards, 1s.

(128) *Books on Nursing.*—We advise *Bookish* to get Luckës' "Lectures on Nursing," 2s. 6d., at our office.

(129) *Nil Desperandum.*—You can take no certificate without training. Your testimonials ought to stand you instead.

(130) *Wing.*—There is no fee; nurses are usually paid £10 the first year. In most hospitals you serve three years for your certificate.

(131) *Names Wanted.*—Would *F. F.* please send address. If *Contented Ones* will send their names we will print their letter. We must insist on all correspondents sending name and address.

(123) *A R.* will gain all information respecting naval nursing by applying to the Medical Director, General Admiralty Offices, Whitehall, Strand, London. Only a few Sisters are employed, and it sometimes takes years before an appointment is obtained, applicants having to take their turn.—*L. S.*

